# The Role of the Microbiota-Gut-Brain Axis in the Development of Alzheimer’s Disease

**DOI:** 10.3390/ijms23094862

**Published:** 2022-04-27

**Authors:** Benita Wiatrak, Katarzyna Balon, Paulina Jawień, Dominika Bednarz, Izabela Jęśkowiak, Adam Szeląg

**Affiliations:** 1 Department of Pharmacology, Wroclaw Medical University, Mikulicza-Radeckiego 2, 50-345 Wroclaw, Poland; d.bdnrz@gmail.com (D.B.); izabela.jeskowiak@umw.edu.pl (I.J.); adam.szelag@umw.edu.pl (A.S.); 2 Laboratory of Genomics & Bioinformatics, Hirszfeld Institute of Immunology and Experimental Therapy, Polish Academy of Sciences, 53-114 Wroclaw, Poland; katarzyna.balon@hirszfeld.pl; 3 Department of Biostructure and Animal Physiology, Wroclaw University of Environmental and Life Sciences, Norwida 25/27, 50-375 Wroclaw, Poland

**Keywords:** neuroinflammation, Alzheimer’s disease, microbiome

## Abstract

Along with the increase in life expectancy in the populations of developed and developing countries resulting from better access and improved health care, the number of patients with dementia, including Alzheimer’s disease (AD), is growing. The disease was first diagnosed and described at the beginning of the 20th century. However, to this day, there is no effective causal therapy, and symptomatic treatment often improves patients’ quality of life only for a short time. The current pharmacological therapies are based mainly on the oldest hypotheses of the disease—cholinergic (drugs affecting the cholinergic system are available), the hypothesis of amyloid-β aggregation (an anti-amyloid drug was conditionally approved by the FDA in 2020), and one drug is an N-methyl-D-aspartate receptor (NMDAR) antagonist (memantine). Hypotheses about AD pathogenesis focus on the nervous system and the brain. As research progresses, it has become known that AD can be caused by diseases that have been experienced over the course of a lifetime, which could also affect other organs. In this review, we focus on the potential association of AD with the digestive system, primarily the gut microbiota. The role of diet quality in preventing and alleviating Alzheimer’s disease is also discussed. The problem of neuroinflammation, which may be the result of microbiota disorders, is also described. An important aspect of the work is the chapter on the treatment strategies for changing the microbiota, potentially protecting against the disease and alleviating its course in the initial stages.

## 1. Introduction

The largest reservoir of bacteria in the human body is the intestinal microbiota, with the greatest abundance in the large intestine. The number of genes encoded by bacterial cells is more than a hundred times greater than the genes of the host cells [1]. The total mass of gut microbes is comparable to the mass of the human brain. The vast majority of microbiota are anaerobic bacteria, including fungi, protists, archaebacteria, viruses, helminths, and other microorganisms. The most numerous types are *Firmicutes* and *Bacteroidetes*, which constitute 80% of microorganisms living in the gastrointestinal tract. They keep the number of other microorganisms under constant control, which has the strongest impact on the host’s health [2,3,4,5,6,7,8,9,10].

Acquiring knowledge about the interaction between the microbiota and the host organism and the impact of microbiota changes on its health is possible mainly thanks to research on animals not colonized by microorganisms, the so-called “germ-free” (GF). These are often rodents bred in sterile conditions. After experimental colonization by specific microorganisms, it is possible to evaluate their influence on various processes in the host organism [5,11]. The microbiota modification is also achieved by administering antibiotics, fecal transplantation, and deliberate infection with specific microorganisms [11].

Knowing the microbiota composition of a given individual with a specific health condition is possible thanks to the development of sequencing the 16S ribosomal RNA (16S rRNA) fragment. Its polymorphism enables the phylogenetic assignment of bacteria detected, e.g., in feces, and the assessment of their influence on the metabolism and functioning of the host [5]. Identifying gut microbes using molecular biology works much better than classical methods. It enables much faster and more accurate assignment to specific phylogenetic groups, especially since most of the bacteria that make up the microbiota are obligate anaerobes [9].

Microbiota in the human body is a dynamic entity and, at the same time, specific to a given person. It changes over time, from the moment a child is colonized shortly after birth, when giving up breast milk in favor of other foods, at puberty, and due to diseases, diet, antibiotics used, stress, aging, and death, even after the end of human life. Both the composition of the microbiota and the number of microbes of individual groups, as well as their mutual proportions, are subject to change [6,9,11,12,13,14,15,16,17,18,19]. These processes may occur without a major impact on the host organism, but they can also lead to serious systemic and metabolic changes, also affecting blood-brain barrier (BBB) permeability [18,20].

## 2. Treatment of Alzheimer’s Disease

Despite over 100 years of research on the pathomechanism of Alzheimer’s disease, there are still no effective drugs. The United States Food and Drug Administration (FDA) has approved only a few compounds for use in people with AD [21]. Most of them concern the oldest hypothesis of this disease—cholinergic, and these drugs are cholinesterase inhibitors [22]. One of the first compounds that inhibited acetylcholinesterase was tacrine, but due to its strong hepatotoxic effect, its use was suspended [23]. The compounds currently used in this field are donepezil and rivastigmine [22,24]. Despite the same mechanism of pharmacological action, they are used interchangeably—if one drug does not work, the second is prescribed after a few weeks. A less commonly used drug from the group of parasympathomimetics is galantamine, which improves nerve conduction and increases the concentration of acetylcholine [22].

If necessary, a second drug, memantine, is added to inhibit another neurotransmitter, glutamate, and thereby inhibit N-methyl-D-aspartate (NMDA) excitotoxicity [25]. However, these drugs only slow AD progression and have not been shown to stop the disease.

Observing the lack of full effectiveness of these drugs, the search for anti-amyloid substances as well as drugs limiting the hyperphosphorylation of the tau protein was then focused on. It should be noted that after an almost 20-year break, in the spring of 2021, a new drug, aducanumab, for use in AD was conditionally approved—with an anti-amyloid effect. However, the adopted theory recognizing amyloid-β deposits as the main source of brain damage seems to be the wrong path in the context of therapy. Attempts to treat with anti-Aβ antibodies resulted in meningitis, an increased risk of upper respiratory tract infections, and increased BBB permeability, manifested by cerebral edema and microhemorrhages [26]. Therefore, it may be assumed that the removal of all amyloid is not an ideal solution. As shown in other studies, amyloid at physiological concentrations offers many beneficial activities, including antioxidant, anti-inflammatory, and antimicrobial [27,28]. Accumulation of excessive amounts may occur in situations of struggle with inflammation, and exceeding a certain level also disturbs the level of microglia, the function of which is to remove it, and therefore chronic inflammation persists.

## 3. Microbiota Changes in Alzheimer’s Disease

The relationship between the microbiota content and the development of Alzheimer’s pathology has been repeatedly observed and demonstrated in animal models.

Sequencing of bacterial 16S rRNA in stool samples showed that transgenic mice (Alzheimer’s disease model) have increased *Rikenellaceae* abundance and decreased Allobacillum and *Akkermansia* abundance compared to the wild-type (wt) mice. The amount of these bacteria correlates with the amount of Aβ42 in the brain [29,30]. In an animal model of Alzheimer’s disease (APP/PS1), a significant increase in the content of bacteria belonging to *Bacteroidetes* and *Tenericutes* and a decrease in the number of *Firmicutes*, *Verrucomicrobia*, *Proteobacteria*, and *Actinobacteria* compared to wild-type mice of similar age were shown. These differences correlate with an increase in amyloid deposition in the brains of APP/PS1 mice [29]. The microbiota composition in APP/PS1 mice and wild-type mice begins to differ at a young age (1–3 months) before the start of amyloid deposition in senile plaques and microglia activation in the plaque area [31]. In the mouse model (Tg-AD) in females (more likely to develop AD), a relationship between cognitive functions and the composition of the microbiota was confirmed. The lower level of cognitive functions was related to decreased abundance of *Ruminococcaceae*, and butyrate levels positively correlated with mental performance [32].

Researchers who conducted a study in 5xFAD mice found the expression of amyloid-β precursor protein (AβPP) not only in the brain but also in intestinal tissue. They suggest that the presence of mutated AβPP and PS1 genes associated with the development of familial AD affects the gut function and thus the microbiota composition [33].

Moreover, dysbiosis induced by broad-spectrum antibiotics in male mice representing an animal AD model influenced the development of inflammation in the nervous system and decreased amyloid plaque deposition [10,30,34]. In germ-free APP transgenic mice (with an expression of the human amyloid precursor protein), microbiota transfer from conventionally bred transgenic mice increased brain Aβ concentration [29].

Alzheimer’s disease in humans also has a very specific microbiota pattern that is significantly different from the control group. A decreased number of *Firmicutes* and *Actinobacteria* and an increased number of *Bacteroidetes* have been observed in patients with AD [35]. In patients with dementia and amyloid deposits, a reduction in the number of butyrate-producing *Eubacterium rectale* and an increase in the number of *Escherichia/Shigella*, defined as pro-inflammatory, have been observed [10,36]. In addition, there is a relationship between the number of bacteria in the types most common among patients and AD biomarkers (e.g., the level of Aβ_42_/Aβ_40_ in the cerebrospinal fluid) [35]. A study on a small group conducted in Japan showed that reduced numbers of *Bacteroidetes* and increased numbers of bacteria identified as “other” are better indicators of AD than traditional biomarkers such as ApoEε4 or VSRAD analysis system [37].

The feces of people with AD and the healthy control group were also examined, showing similar changes as in mice—a reduced number of *Firmicutes* and *Bifidobacterium*, and an increased number of *Bacteroidetes*. The differences in the microbiota correlated with the levels of AD biomarkers in the cerebrospinal fluid (CSF) [35]. Interestingly, there was a significant correlation between the incidence of irritable bowel syndrome (IBS) in patients and the risk of dementia. It has been observed that if IBS is diagnosed before the age of 50, the risk of developing dementia in the future increases [38]. Disturbance of the intestinal microflora may affect not only the accumulation of amyloid in extracellular deposits in the brain but also the reactivity of the glial located in senile plaques. It was observed that long-term administration of broad-spectrum antibiotics to APP_SWE_/PS1_ΔE9_ mice (AD model) regulated amyloidosis, decreased circulating pro-inflammatory cytokines, and influenced the microglia morphology [34].

The gut microbiome influences the integrity of the blood-brain barrier and the neuroimmune response, including through bacterial metabolites, particularly SCFAs [9,16,18,39]. In addition, some bacteria such as *Lactobacillus Plantarum*, *E. coli* Nissle or *Bifidobacterium infantis* can strengthen the barrier constituted by the intestinal wall [30,39]. The question remains whether the changes in the microbiota can precisely and selectively influence the aspects mentioned above.

## 4. The Microbiota-Gut-Brain Axis

Such numerous and diverse microorganisms inhabiting the interior of humans play a significant role in digesting and supplying the host with nutrients and necessary metabolites. Still, they can also pose a serious threat if the protective barrier of the intestinal epithelium is exceeded [5,8].

However, microorganisms do not have to escape the lumen of the digestive tract to influence the processes of the host, such as, e.g., metabolism, behavior, and the immune system, as shown by the functioning of the “microbiota-gut-brain axis”. Neurotransmitters, the transmission of stimuli through the vagus nerve, secreting short-chain fatty acids (SCFA), microRNA (miRNA), small non-coding RNA (sncRNA), and other active molecules are responsible for the contact of the gut microbiota with the central nervous system. The second factor influencing the communication of the gut microbiota with the central nervous system (CNS) is the permeability of the blood-brain barrier and intestinal wall, which increases with age [2,6,7,8,10,16,17,40,41].

### 4.1. Neurotransmitters

Microbiota stimulates neurotransmitters and their precursors. This specific communication on the microbiota-gut-brain axis is called the neuroendocrine system [42]. Bacteria produce their metabolites and can trigger signals that affect the synthesis and conduction of neurotransmitters. This information can be transmitted locally in the gut or routed to the brain through the vagus nerve [43]. Serotonin, gamma-aminobutyric acid (GABA), glutamate, and dopamine are neurotransmitters that do not cross the blood-brain barrier but are synthesized thanks to local precursors in the central nervous system [44]. For a given neurotransmitter to be synthesized, it needs a precursor that often comes from the digestive system. These precursors pass from the digestive system into the blood and then cross BBB and are taken up by the cell, synthesizing the neurotransmitter. Often the transformation of the precursor takes place in several steps in which enzymes are also involved [44].

Serotonin (5-HT) is a neurotransmitter, the vast majority of which (about 90%) is produced by enteroendocrine cells [2,45]. It has been shown that short-chain fatty acids produced by bacteria can induce the production of serotonin in the intestine, and in the absence of intestinal microbiota, serum 5-HT levels and its metabolites and precursors in urine and intestinal contents are reduced [45]. The main precursor to serotonin is tryptophan. In the absence of gut microbiota in the early stages of development of GF mice, increased serum levels of tryptophan and increased levels of 5-HT in the hippocampus were observed in adult animals. However, this effect was reversible upon colonization with probiotic *Bifidobacterium infantis* [2,16,17]. Tryptophan is produced by bacteria that inhabit the gut and has been shown to pass from the periphery to the central nervous system across the blood-brain barrier [45]. For example, *Clostridium sporogenes* and *Ruminococcus gnavus* produce tryptamine, stimulating enteroendocrine cells to produce 5-HT. It has been shown that tryptamine can cross the BBB, but it is uncertain whether gut-derived tryptamine influences neurological functions in the same way [45]. The same applies to tyramine, which is produced, among others, by *Levilactobacillus brevis* or a species belonging to *Enterococcus*, whose direct effect on the nervous system has not been demonstrated so far [45].

Other neurotransmitters that change in germ-free mice are GABA and dopamine. Their serum concentration decreases, accompanied by changes in the concentration of precursors to these neurotransmitters in the intestine [45]. GABA, the main neurotransmitter with inhibitory properties on signal transduction, is produced by bacteria belonging to *Lactobacillus* and other species of the *Bifidobacterium* genus [6,17]. Disturbances in GABA-related conduction pathways have been demonstrated, among others, in cognitive disorders such as Alzheimer’s disease (AD) [6]. Although no data suggest that serotonin or GABA cross BBB, changes in their levels in the CNS may result from the action of these neurotransmitters on the vagus and peripheral nerves or changes in the concentration of their precursors [45].

Thanks to dopamine signaling through the gut-brain axis, the brain reaches, among others, for information in the satiety center so that the reward center can be activated. Dopamine is a precursor to other catecholamines—noradrenaline and adrenaline. Noradrenaline is associated with activating the arousal response in times of danger or wakefulness. Still, recent reports suggest that it also plays an important role in cognitive and memory functions. Studies in an animal model have shown that the level of noradrenaline in the cecal in mice lacking the gut microbiota is reduced [46,47]. In contrast, by colonization with *Clostridium* species, this level can be increased [48]. It was also noticed that the metabolism and circulation of noradrenaline and adrenaline in the GF mice are faster than after colonization with the microbiota, which suggests that the total amount of these catecholamines in the systemic circulation is lower than in the presence of microorganisms [48].

According to the theory of NMDA receptor dysfunction, it was tested whether glutamate metabolized by gut bacteria could improve cognitive functions in patients with AD. It has been observed that the microbiota of neuropsychiatric disease patients containing *Bacteroides vulgatus* and *Campylobacter jejuni* influence glutamate metabolism and, consequently, a decreased level of its metabolite—2-keto-glutaramic acid was recorded [49]. Additionally, *Corynebacterium glutamicum*, *Brevibacterium lactofermentum*, and *Brevibacterium avium* influence the conversion of l-glutamate to d-glutamate, which may affect cognition in patients with dementia. Moreover, probiotic *Lactobacillus* strains can synthesize glutamate [49]. Increased levels of D-serine have been shown to be associated with poorer cognitive functions such as remembering words, understanding words, and finding words. At the same time, the increase in D-alanine caused greater behavioral difficulties. In contrast, the decrease in D-glutamate level affected the disorders in naming objects and fingers, carrying out orders, and understanding [50]. Reduced levels of D-glutamate in the blood plasma have been observed in patients with cognitive impairment [51]. At the same time, increased levels of glutamate were observed in the cerebrospinal fluid (CFS) compared to healthy people. Moreover, glutamate levels were inversely correlated with other AD disease biomarkers assessed in CFS [52]. Based on the results confirming the effect of D-glutamate on cognitive dysfunction in the analysis of stool samples of both animals and humans, it has been confirmed that it may be a potential biomarker for distinguishing the severity of the disease in mild cognitive impairment and AD [53].

Undoubtedly, the intestinal flora plays a significant role in producing neuromediators. However, more research is still needed to explain the mechanisms involved.

### 4.2. Short-Chain Fatty Acids

Short-chain fatty acids (SCFAs) secreted by the gut microbiome include butyrate, propionate, and acetate produced by bacteria of *Clostridium*, *Eubacterium*, and *Butyrivibrio*.

In studies, sodium butyrate showed a positive effect on learning and memory in transgenic mice that were AD models, even in the late stages of the disease [18,54]. Butyrate is a molecule with neuroprotective properties and it positively affects the brain’s functioning. Additionally, it is an important energy substrate and increases the mitochondrial production of ATP [18]. It can also restore the integrity of the blood-brain barrier [45]. Further, it reduces intestinal permeability and exhibits anti-inflammatory properties by inhibiting the secretion of pro-inflammatory cytokines by cells of the immune system [19].

Acetate, another common SCFA, can cross the BBB, inducing a feeling of satiety and changing the levels of neurotransmitters. In addition, it affects the functioning of the microglia. Finally, it reduces the permeability of the BBB, which reduces the exposure of the central nervous system to active compounds originating from outside the CNS [18,45]. This is of particular importance when the permeability of the intestinal wall increases with age [3,16] and under the influence of stress [2,16].

Propionate protects the blood-brain barrier by reducing the influence of pro-inflammatory and oxidative factors [55].

### 4.3. Vagus Nerve Conduction

The vagus nerve regulates the work of the middle and lower digestive system by influencing its mobility, mucosal function, blood flow, etc. However, signaling is not only one way; it also allows intestinal stimuli to affect brain function. Vagus nerve conduction appears to be essential for the systemic effects of probiotic therapy. For example, using a probiotic strain of *Lactobacillus rhamnosus* (JB-1) reduces behaviors related to anxiety and depression. It also reduces the release of corticosteroids in response to stress and changes in GABA receptor expression, only in people with an intact vagus nerve. However, the vagus nerve is not the only communication between the gut microbiota and the central nervous system. Behavioral changes following the use of broad-spectrum antibiotics were also observed in vasectomized mice [2,17,43,45,56].

### 4.4. Blood-Brain Barrier Permeability

The permeability of the blood-brain barrier (BBB), as well as the permeability of the intestinal wall, increases with age, which raises the exposure of the central nervous system to potentially harmful particles produced by the physiological microbiota and its accompanying pathogens [3,4,16,18,45,57]. Additionally, the integrity of the BBB depends on the proper composition of the intestinal microbiota, which may be related to the SCFAs they produce [16,45,58]. Reduced butyrate levels have been observed in the elderly, probably caused by insufficient fiber intake in the daily diet [12]. As the microbiota changes with age, the production of SCFAs is also reduced [18]. With a decrease in the number of *Bifidobacteria* and *Firmicutes* and an increase in the number of *Bacteroidetes* and *Proteobacteria*, including *Enterobacterales*, the level of diversity of microorganisms inhabiting the gastrointestinal tract decreases [12,19].

### 4.5. Bacterial Amyloids

Bacterial amyloids enter the brain with increased permeability of the blood-brain barrier and may also influence the development of Alzheimer’s disease [57]. The most studied curli fimbriae, produced by *Escherichia coli* as a component of their biofilm, show a biochemical similarity to Aβ. In addition, the host immune response to curli (especially its major subunit—CsgA) produced by *Salmonella* and *E. coli* closely resembles the response to toxic Aβ oligomers (by activation of TLR2) [59,60,61,62]. Moreover, these molecules of bacterial origin can, together with amyloid-β (Aβ) produced by the host, co-create senile plaques [10,63]. Due to their similar structure, bacterial amyloids can induce human proteins to adopt the pathological β-sheet structure [30].

### 4.6. Fragments of Bacterial Cells

Other bacterial molecules, such as genetic material and fragments of their cell walls, including peptidoglycans, flagellin, and highly pro-inflammatory lipopolysaccharide (LPS), may also cross the leaky blood-brain barrier [18,30,64]. Moreover, LPS, like bacterial amyloids, contribute to the formation of senile plaques [30,40,64,65]. This may directly impact the development of Alzheimer’s disease [57,64].

According to one hypothesis, the main cause of Alzheimer’s disease is the excessive accumulation of amyloid-β in senile deposits in the brain. Aβ, in many ways, resembles the biomolecules known collectively as antimicrobial peptides (AMPs), among which aggregation is a normal physiological response [59,66,67]. They are broad-spectrum antibiotics produced by the human body and are modulators of the immune response [26,66]. The potential mechanism of their action is based on the ability to aggregate and integrate with cell membranes (Aβ, like LL-37 belonging to AMPs, has a heparin-binding fragment). Then they create pores, causing an uncontrolled flow of substances through the membrane and, as a result, cell death [26,67,68,69]. However, Aβ, until recently considered only harmful, exhibits several positive properties at physiological concentrations in the brain, including neurotrophic, antioxidant, or antibacterial [27,63,70]. There is also a theory that amyloid-β protects the brain from harmful particles crossing the leaky blood-brain barrier by binding them into insoluble deposits [26,67]. Aβ may also be involved in sealing BBB—AD amyloid plaques form around the capillaries [26].

The synthetically produced amyloid-β shows antibiotic activity against Gram-positive and Gram-negative bacteria and *Candida albicans* fungi [66,71,72]. Moreover, the presence of Aβ deposits in the brain is not a pathognomonic feature of AD. However, amyloid plaques in the brain have been demonstrated in diseases such as neuroborreliosis, HIV-related dementia, and chlamydia infection [57,73]. In a study measuring the antimicrobial properties of human frontal lobe samples, materials from AD patients were significantly more effective than materials from the control group. Furthermore, this effect can be largely reduced by the use of anti-Aβ antibodies [26,66]. In turn, in transgenic mice infected with *Salmonella typhimurium* by intracerebral injection, 5xFAD rodents displaying consistently high amyloid-β expression lived significantly longer than APP-knockout mice (lacking Aβ precursors) [67]. Similarly, *Caenorhabditis elegans* transgenic nematodes expressing Aβ42 (GMC101) had reduced mortality from *C. Albicans* infection when compared to nematodes that do not produce human amyloid (CL2122) [67].

## 5. Infections and the Development of Alzheimer’s Disease

The microorganisms that are part of the microbiota can contribute to the development of Alzheimer’s disease. Previous infections and pathogens encountered are also potentially important. For example, multiple infections within four years have doubled the risk of developing Alzheimer’s disease (Table 1) [63,65].

Infection with *Porphyromonas gingivalis*, which causes periodontal disease, is associated with a significant increase in the risk of developing AD [3,64,74,75,76]. This may be due to a change in the gut microbiota composition in people affected by the infection in the oral cavity. After administration of *P. gingivalis*, a decrease in the percentage of *Bacteroidetes* and an increase in the number of *Firmicutes* in stool samples were observed [18]. In addition, the bacteria that cause periodontitis can also be the source of significant amounts of pro-inflammatory molecules, such as LPS, peptidoglycan, flagellin, or bacterial DNA [18,64].

HSV-1 infection increases the risk of developing AD among APOE4 allele carriers [63]. Activation of a dormant virus in nervous tissue can induce inflammation, amyloidogenesis, and neurodegeneration. Consequently, it may accelerate or increase the likelihood of developing AD in predisposed individuals [3,6,57,77]. In turn, the herpes B virus glycoprotein shows significant similarity and structure homology to amyloid-β and may enhance amyloidosis [57]. There was also a specifically increased expression of non-coding, pro-inflammatory, immunomodulatory miRNA-146a in HSV-1-affected individuals. Overexpression of this miRNA was also observed in the brains of AD patients [3,78].

Infection with C. *pneumoniae* in carriers of the APOE4 allele also increases the risk of developing AD [63]. Moreover, chlamydia, amyloid deposits, and neurofibrillary tangles occupy the same places in the brain, and *C. pneumoniae* antigens (both intra- and extracellular) have been detected in the frontal and temporal lobes of patients with AD [3,57]. In a study in mice, inhalation infection with *C. pneumoniae* stimulated amyloid-β deposition [57]. It is also worth noting that most AD patients do not die of the disease itself but of pneumonia [3,57].

Other infectious agents associated with AD development are *Spirochaetales*, *Helicobacter pylori*, *Candida glabrata*, *Toxoplasma gondii*, HIV and CMV viruses [3,30,57,61,65,72]. In addition, vaccination against influenza and other diseases, including DTP vaccines, significantly reduces the risk of developing AD [63,64].

### Neuroinflammation

**Figure 1:**
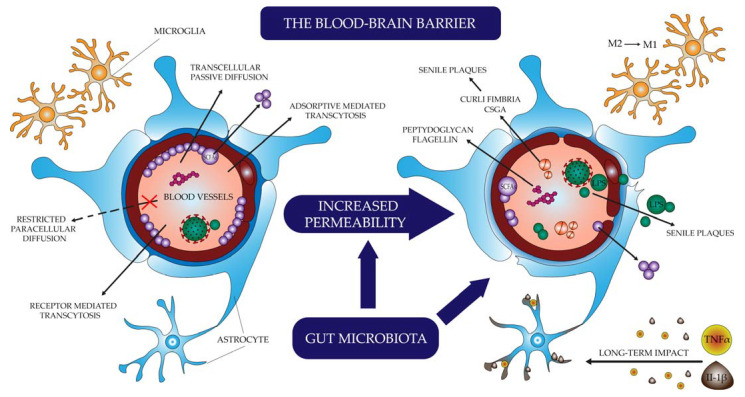
The process of neuroinflammation.

The biological response that can link infection, changes in the microbiota, molecules secreted and produced by microorganisms, and changes in the brain leading to the development of AD symptoms is inflammation, more specifically neuroinflammation (Figure 1) [70,79]. In amyloid deposits, an increased number of astrocytes and microglia and elevated levels of pro-inflammatory cytokines are observed—Alois Alzheimer himself is the first to mention the inflammatory component of the disease picture [36,70,79,80].

Microglia are cells derived from a monocyte lineage capable of phagocytosis and the production of cytokines. When activated, they may be in the M1 state (pro-inflammatory, promoting the secretion of active molecules) or M2 state (anti-inflammatory, promoting phagocytosis) [39,79,81,82]. Depending on the phenotype and the circumstances accompanying activation, they may have a protective or destructive effect on neuronal cells. Foreign particles reaching the brain as a result of infection or through leakage of the blood-brain barrier, as well as debris of cells damaged by pathogens and other destructive factors, stimulate microglial cells to transition to the state defined as M1. They act through pro-inflammatory cytokines such as TNF-α and IFNγ and are activated through Toll-like receptors (TLR) stimulation [39,79,83]. Amyloid-β itself, via TLR and RAGE, also stimulates the activation of microglia M1 [8,79,83]. It is a physiologically short-lived phenotype designed to fight the infectious agent and repair tissue. In AD, however, there is chronic inflammation caused by continuous stimulation with pro-inflammatory agents. This causes the neurodegenerative action of microglia with uncontrolled production of pro-inflammatory cytokines through NF-κB activation and ineffective phagocytosis, preventing effective clearance and promoting β-amyloid production and deposition. As a result, it disrupts the differentiation and maturation of precursor nerve cells, inhibits the formation of new synapses, and reduces the plasticity of neurons [8,57,64,79,80,82].

It is worth noting, however, that activation of Toll-like receptors at different stages of the disease may have other effects. In the initial stages, it has been shown to reduce amyloid aggregation. In the later stages, it leads to the development of inflammation and neurotoxicity [57,84]. Early microglia stimulation by TLR2 and TLR4 leads to cell transition to an M2 anti-inflammatory state associated with reduction of amyloid-β plaques, improved neuronal plasticity, and enhanced cognitive ability. Stimulation with TLR2 and TLR4 ligands at an early stage of pathology development in rats receiving amyloid-β resulted in a therapeutic effect that improved cognitive abilities and reduced amyloid deposits [84]. However, soluble Aβ oligomers alone cannot activate microglia before aggregation into plaques and they are neurotoxic [84,85,86]. At a later stage, the TREM2 receptor protein on the microglia surface reduces activation by TLR2 and TLR4, acting in a neuroprotective and anti-inflammatory manner, playing a protective role in the pathogenesis of AD. A meta-analysis of several genome-wide association studies (GWAS) has shown a connection between a mutation in the gene encoding TREM2 resulting in an R47H substitution and an increased risk of developing AD [82,83].

In APP germ-free transgenic mice, microglia show reduced production of pro-inflammatory cytokines [17,29,41]. At the same time, the concentration of Aβ in their brains is lower than in colonized mice [29]. Furthermore, transgenic animals incapable of producing pro-inflammatory Il-1β show reduced amyloid plaques by microglia activation, promoting phagocytosis [29].

Other inflammatory cells—astrocytes—play a crucial role in maintaining the integrity of the BBB. They are capable of detecting Aβ deposits and respond by producing pro-inflammatory cytokines. At the same time, exposure of astrocytes to the continuous action of TNF-α and Il-1β has a destructive effect on the maintenance of the blood-brain barrier through which more inflammation-inducing molecules pass [79,82].

Additionally, generalized inflammation inhibits β-amyloid outflow from the cerebrospinal fluid [87]. Risk factors for developing Alzheimer’s disease, such as type 2 diabetes, obesity, and metabolic syndrome, are characterized by low-grade chronic generalized inflammation, which may also be associated with disturbances in the microflora composition [20,39,82]. Increased levels of CRP and Il-6 in the serum—proteins related to the inflammatory process in the body—are observed years before the development of dementia [65].

## 6. Prevention and Treatment Strategy for Alzheimer’s Disease

### 6.1. Studies in Animal Models

Knowledge about the effects of colonization with specific strains of bacteria is constantly expanding, and the possibilities of creating targeted probiotics are getting closer. However, most of the knowledge gained is based only on animal research. Studies on mouse models have confirmed that microbiota modulation positively affects neuronal pathways. In behavioral experiments on mice, it was shown that taking probiotics slows the progression of Alzheimer’s disease and influences the process of memory consolidation in mice.

Research on an animal model shows that modulation of the intestinal microbiota by supplementation with probiotics for several months effectively reduces gliosis and eliminates amyloid-β aggregation. Qualitative improvement of the microbiome and the release of anti-inflammatory, positive intestinal microbiota metabolites determine beneficial changes, reducing pro-inflammatory markers characteristic of Alzheimer’s disease and significantly reducing systemic inflammation. Moreover, improvements in cognitive functions and memory in the mice model were noted. *Bifidobacterium breve* has been shown to reduce inflammation in the nervous system caused by amyloid-β aggregation. In turn, administering a mixture of strains from the *Bifidobacterium* and *Lactobacteriaceae* families to mice in the early stages of AD reduced the accumulation of toxic amyloid. This inhibited the disease progression [88,89].

A probiotic blend of eight Gram-positive bacteria strains called VSL#3 has been thoroughly tested. In animals subjected to this treatment, changes in the microbiota composition were observed, accompanied by improved synaptic conductivity, a decrease in microglia activity, and changes in the expression of genes responsible for neuronal plasticity and inflammation [16]. In turn, supplementation with probiotic *Bifidobacterium breve* strain (NCIMB 702258) led to an increase in the concentration of arachidonic acid (ARA) and docosahexaenoic acid (DHA) in the brain. They play an important role in neurogenesis and nerve conduction, protect against oxidative stress, and impact memory and learning ability [43].

Bonfili’s research group treated 3xTg-AD mice for 4 months with the preparation of lactic acid bacteria and bifidobacteria (SLAB51). AD mice treated with probiotics improved cognitive performance compared to the untreated group, but no significant differences were observed in wild-type (wt) mice. Probiotics changed the composition of the intestinal microflora, reducing the level of inflammatory cytokines and the amount of Aβ, which resulted in improved behavioral performance. Additionally, reduced concentrations of *Tenericutes*, *Cyanobacteria*, *Anaeroplasmatales*, and *Anaerostipes* were present in 3xTg-AD mice compared to wt mice [90]. Similar results in male C57BL/6J mice were obtained by a Chinese research team who stated that *Bifidobacterium breve* CCFM1025 and WX have a beneficial effect on cognitive functions and delay the progression of AD by modulating the intestinal microbiome [91]. Moreover, in a study by Kaur et al., in App^NL-G-F^ female mice treated with a mixture of *Lactobacillus* and *Bifidobacterium*, a reduction of Aβ plaques and memory improvement were observed [92].

Furthermore, Ma et al. [93] administered probiotic bacteria such as *Bifidobacterium* and *Lactobacillus* for 5 days to SPF C57BL/6 male mice with LPS-induced systemic inflammation, ampicillin-induced intestinal dysbiosis, and cyclophosphamide-induced immunosuppression. Probiotics, through their synergistic effects, alleviated intestinal microflora disturbances. They improved the functioning of the immune system at the level of the intestinal mucosa, and thus reduced inflammation (caused, among others, by the release of pro-inflammatory cytokines) and cognitive impairment [93]. In a study on male C57BL/6 mice, Jang et al. demonstrated that *Lactobacillus johnsonii* alleviated intestinal dysbiosis, colitis, and depression induced by ampicillin and reduced the level of anxiety assessed in behavioral tests [94]. In addition, it has been found that *Lactobacillus paracasei* PS23 (LPPS23) can delay age-related cognitive decline by modulating brain-gut communication and reducing inflammation. The positive effect of LPPS23 was demonstrated in tests on male and female aging-accelerated mice (SAMP8) by assessing their appearance and behavior as well as inflammatory cytokine levels [95].

Other studies on albino adult mice with AD induced by injection of streptozotocin show that an alteration in the gut microbiome due to insulin resistance is a key risk factor for developing Alzheimer’s disease. What is more, insulin resistance is also increased in the inflammation. Probiotics fermentation technology (PFT) kefir, which was served to mice, contained a combination of *Lactobacillus* and multiple yeast strains (*Kazachstania*). Due to the reduction of inflammation and insulin resistance, PFT decreased Aβ42 protein accumulation and tau protein hyperphosphorylation [96,97].

Moreover, probiotics and prebiotics such as mannan oligosaccharide (MOS) change the gut microbiota content and increase the formation of neuroprotective SCFAs. After 8 weeks of therapy in 5xFAD transgenic mice, Aβ accumulation in the hippocampus and cortex significantly decreased, and thus their cognitive functions improved and anxiety behaviors decreased [98]. Another research team performed fecal microbiota transplantation (FMT) from 5xFAD mice into C57BL/6 mice and vice versa. The obtained results showed that FMT from healthy mice reduced neurogenesis in the hippocampus responsible for memory impairment in mice suffering from AD [99].

Téglás et al., in the study on APP/PS1 transgenic mice, showed that the implementation of probiotic supplementation with *Bifidobacterium longum* and *Lactobacillus acidophilus* strains enriched with omega-3 fatty acids and B vitamins connected with physical exercises improved exploratory ability and spatial memory [100].

The latest research results on mouse models clearly show that oral administration of probiotic bacteria such as bifidobacteria and lactic acid bacteria in AD disease reduces cognitive deficits by reducing inflammation or the amount of Aβ accumulated in the brain structures.

### 6.2. The Influence of the Diet

A properly selected diet can significantly impact the composition and quality of the intestinal microbiota and its metabolites [10,15,17]. For example, SCFAs are produced by fermentation from dietary fiber. In the conducted studies, a diet rich in fiber alone is enough to improve cognitive abilities in healthy children significantly [18]. Conversely, reduced fiber consumption leads to a decrease in the diversity of the intestinal microbiota, which in turn is associated with a deterioration of cognitive abilities [15].

There is a correlation between cultural preferences and eating habits and this has led to some hypotheses about inhibiting Alzheimer’s disease progression or longevity. An example is France, an important producer and a country where people enjoy consuming red wine rich in resveratrol, which has strong antioxidant activity, thus counteracting oxidative stress. Studies support the theory that moderate red wine consumption may reduce the risk of developing AD. In addition, the diet used in Mediterranean countries due to omega-3 fatty acids is also considered to have neuroprotective effects. It should be noted that the countries of the Mediterranean basin differ significantly in habits and lifestyles from Northern Europe and Western countries. Hence, fewer daily stressors may play a key role in influencing the quality and length of life [101,102].

A study analyzing the impact of the Mediterranean diet on changes in the intestinal microbiota showed beneficial reorganization changes in bacterial strains. With the use of the diet, the amount of *Escherichia coli* decreased, while the content of the genus *Bifidobacterium* increased. In addition, an increase in the amount of acetate has been observed, which plays an important role in sealing the gut wall and the blood-brain barrier (BBB). When examining the antagonistic dependence of using a low-quality fast-food diet, a reduction in the beneficial bacterial microflora and the amount of butyrate was observed. A high-fat diet increased the number of *Streptococcaceae*, especially *Streptococcus* [103,104] and led to elevated blood levels of LPS and generalized inflammation [39,105]. A load of this endotoxin may result from reduced expression of proteins responsible for sealing the intestinal wall, a decrease in the thickness of the mucosa, and disturbances in the production of antibiotic peptides in the case of a high-fat diet [105].

In Asian countries, turmeric (*Curcuma longa* L.) has long been widely used. For example, turmeric root has been used as a wound-healing agent. A distinctive feature of Asian cuisine is enriching dishes with this spice. Curcumin has a strong antioxidant and anti-inflammatory effect. It protects mitochondria against oxidative damage, regulates the level of glutathione in cells, and relieves neuroinflammation [106,107,108].

Diet quality is of particular importance in the context of the elderly. With age, the problem related to the digestibility of nutrients increases. It is conditioned by many factors, such as salivation, difficulty swallowing, leaky gut, or change in the food absorption surface due to the degradation of the intestinal villi. Pharmacotherapy used by the elderly also affects the appetite and reduces food digestibility. It is often associated with an increase in gastric pH and disturbance of its secretion. Non-steroidal anti-inflammatory drugs (NSAIDs) can be used in AD to prevent chronic neuroinflammation, but this often causes small intestine ulceration [109,110,111,112]. At the same time, when used as a preventative drug, long-term use of NSAIDs is associated with a significantly reduced risk of developing AD [80,112].

The elderly are characterized by a reduced ability to perceive the sharpness of flavor and smell. The consequence is a lack of appetite and deterioration in the quality of the selected food. Older people sometimes skip meals or specific products that, for example, are difficult to chew. As a result, this often leads to micro/macroelement deficiencies and malnutrition [113].

### 6.3. Probiotic Therapy of AD in Humans

There is still insufficient evidence for the effectiveness of probiotic therapy and its positive effects on cognitive function in Alzheimer’s disease. According to a meta-analysis conducted by Deng et al., [114] experiments with patients so far are rather controversial and inconclusive. Nevertheless, the authors of the meta-analysis identified five studies that found significant improvement in cognitive performance after probiotic therapy. This analysis concluded that the potential beneficial effect of probiotics is based on the reduction of anti-inflammatory markers and oxidative processes [114].

In a study conducted on AD patients, people receiving for 3 months a probiotic mix of *Lactobacillus acidophilus, Lactobacillus casei, Bifidobacterium bifidum*, and *Lactobacillus fermentum* obtained significantly better results in the Mini-Mental State Examination (MMSE) test used to assess dementia [42]. Considering the observed reduced diversity of the intestinal microbiota in people with AD, probiotic supplementation may bring promising effects. Furthermore, probiotics have shown immunomodulatory activity, and this treatment strategy supports the immune system’s response. The production of cytokines; the improvement of the function of natural killer cells, macrophages, granulocytes, or T lymphocytes; and the systemic antibody response are observed.

In another study, elderly people were administered probiotic strains such as *Bifidobacterium bifidum* BGN4 and *Bifidobacterium longum* BORI. The patients were assessed for cognitive function, stress level, and the levels of the brain-derived neurotrophic factor BDNF. After 12 weeks of treatment, a reduction in the microbiota of *Eubacterium*, *Allisonella, Clostridiales*, and *Prevotellaceae*, which are considered to be responsible for inflammation, was observed. At the same time, based on the completed questionnaires from Consortium to Establish a Registry for Alzheimer’s Disease (CERAD), an improvement in cognitive functions and a reduction in stress levels were found. Furthermore, an increase in the level of BDNF in the blood serum was observed, which correlated with the reduced content of *Eubacterium* and *Clostridiales* strains [115].

Sanborn et al. also observed that a 3-month supplementation with the *Lactobacillus rhamnosus* strain in patients over 50 with cognitive impairment improves these functions, which was confirmed by the NIH Toolbox Cognitive Function Battery (CFB) neuropsychological test [116].

An interesting study was conducted by the Tamtaji team, where patients with AD were divided into two groups. The first one received a mixture of probiotics (*Lactobacillus acidophilus*, *Bifidobacterium bifidum*, and *Bifidobacterium longum*) with 200 μg of selenium twice a day for 3 months, and the second group took a placebo. In the group of patients taking the probiotics, an improvement in cognitive functions, a decrease in the C-reactive protein level, and an increase in the total glutathione level were observed [117].

High hopes are associated with the possibility of transferring microbiota from healthy people to treat dysbiosis and improve the functioning of both the gastrointestinal tract and the brain [18]. The efficiency of such a transfer has been repeatedly demonstrated in animal models of various neurodegenerative diseases. In addition, there are reports of a positive effect of this treatment in patients with autism, multiple sclerosis, and Parkinson’s disease. However, success in AD patients has not been achieved so far [30].

Another interesting concept of the microbiota modification method is the use of nematodes. The idea is based on the co-evolution of the human microbiome and these parasites, and the ability of worms to influence the composition of the intestinal microbiota through secreted antibiotic molecules, induced immune response, or changes in the gut environment [18].

Pathological changes in Alzheimer’s disease can be prevented by intervening with probiotics and prebiotics, thus achieving homeostasis on the gut-brain axis. The implementation of probiotic supplementation gives an innovative view on the treatment and support of therapy in neurodegenerative diseases [118,119].

## 7. Conclusions

**Figure 2:**
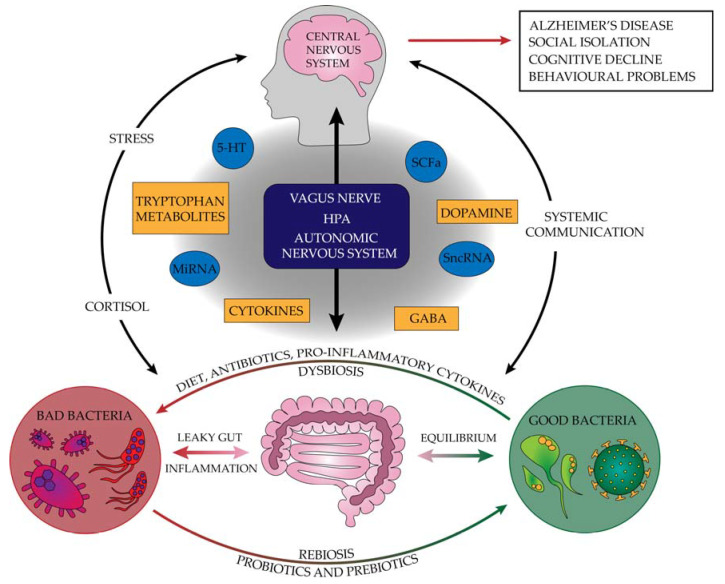
The pathogenesis of neurodegenerative diseases and factors protecting against their incidence that inhibit their progression (5-HT—serotonin, GABA—gamma-aminobutyric acid, HPA—hypothalamus–pituitary–adrenal, miRNA—microRNA, SCFAs—short-chain fatty acids, sncRNA—small non-coding RNA).

Alzheimer’s disease is becoming more common. This is due to the development of medicine, which translates into an extended life span. Until now, the two most widely recognized causes of this disease have been amyloid deposits and neurofibrillary tangles. However, research into anti-Aβ drugs has found that amyloid also has several beneficial effects. The complete removal of amyloid can cause, among others, swelling and bleeding in the brain. When looking at the positive aspects of Aβ, it should be noted that it has antibacterial, antioxidant, and neurotrophic properties [27]. The causes of the disease should be examined by looking at the body as a whole, not just the nervous system or one organ—the brain. More and more evidence points to the influence of the digestive system and intestinal microbiota disorders on the slow changes in the brain and the subsequent development of Alzheimer’s disease. During life, ongoing inflammation in the body may also translate into the development of neurodegenerative disorders. Figure 2 summarizes the pathogenesis of neurodegenerative diseases and shows factors that protect against their incidence and inhibit their progression. In turn, taking NSAIDs in small doses over a longer period shows a positive protective effect against disease development. At the same time, with the occurrence of clinical symptoms, this effect is no longer noticeable [111,112].

Several studies have shown the difference in the composition of the microbiota of healthy people and patients with neurodegenerative diseases such as dementia or AD. Improving the gut microbiota could be a new direction in research into treating neurodegenerative diseases, including AD. Probiotics, prebiotics, and the Mediterranean diet can help improve cognitive functions and inhibit the development of dementia [120,121,122]. It seems, however, that we still need to focus on a thorough understanding of the pathomechanism of Alzheimer’s disease while looking for effective nutritional or pharmacological therapies.

## Figures and Tables

**Table 1:** Types of microorganisms causing the infection and their effects on the development of the disease.

Type of Microorganism	Effect
*Porphyromonas gingivalis*	Reduced amount of *Bacteroidetes* Increased amount of *Firmicutes* Induction of amyloid-β aggregation caused by bacterial amyloids and pro-inflammatory particles
*C. pneumoniae*	Induction of amyloid-β aggregation
*Spirochaetales*	Increased risk of AD progression
*Helicobacter pylori*	Increased risk of AD progression
*Candida glabrata*	Increased risk of AD progression
*Toxoplasma gondii*	Increased risk of AD progression

## Data Availability

Not applicable.
